# An all-arthroscopic light bulb technique to treat osteonecrosis of the femoral head through outside-in fashion without distraction: A case report

**DOI:** 10.3389/fsurg.2022.944480

**Published:** 2022-10-13

**Authors:** Hua-zhang Xiong, Yu-hong Deng, Ying Jin, An-hong Wang, Song Hong

**Affiliations:** ^1^ Department of Orthopedic Surgery, Affiliated Hospital of Zunyi Medical University, Zunyi, China; ^2^ Department of Orthopedic Surgery, People’s Hospital of Yinjiang Tujia and Miao Autonomous County, Yinjiang, China

**Keywords:** arthroscopy, distraction, treatment, femoral head, osteonecrosis, case report

## Abstract

The technique of distraction has been widely used in hip arthroscopy for opening joint spaces. However, an all-arthroscopic light bulb technique through outside-in fashion without distraction has not been reported for the treatment of osteonecrosis of the femoral head (ONFH). A 29-year-old man was admitted to our department with hip pain and limited range of motion (ROM) in both hips over 4 months. X-rays, computed tomography (CT), and magnetic resonance imaging (MRI) showed a mixed appearance, including sclerosis and cysts on the anterosuperior site of the bilateral femoral heads. The patient had an 11-year history of liquor intake. In addition, no other pathologies were found before the operation. After diagnosing bilateral ONFH (stage II) according to the Ficat classification, the patient underwent an all-arthroscopic light bulb technique through outside-in fashion without distraction because of failing conservative treatment. At the 2-year postoperative follow-up, the patient had neither pain nor limitation of ROM. The postoperative x-ray, CT, and MRI revealed a well-healed area of the previous bone grafting in the bilateral femoral heads. An all-arthroscopic light bulb technique through outside-in fashion without distraction can be a feasible method for the treatment of early-stage ONFH. This case reminds us that distraction- and perforation-related complications may be avoided in patients with ONFH without the concomitant pathologies of the central compartment.

## Introduction

Osteonecrosis of the femoral head (ONFH) is considered a source of hip disability and pain. It frequently occurs in patients between 30 and 50 years. The range of ONFH incidence is between 8.9/100,000 and 28.9/100,000 (1). Vascularized or non-vascularised bone grafts are considered a useful method to defer or delay total hip arthroplasty (THA) for hip-preserving procedures of early-stage ONFH, especially in young patients (2, 3). Recently, fast-track surgery (FTS) has become the goal for managing hip lesions. FTS is an evidence-based multimodal approach that can shorten rehabilitation time and improve clinical results, including reductions in both mortality and morbidity, thereby significantly decreasing the time taken to reach hospital discharge and optimizing patient satisfaction (4). Necrotic bone removal and autologous bone grafting procedures (light bulb technique) through minimally invasive open and arthroscopic fashion have become increasingly popular with patients and doctors for treating ONFH (5–7). To our knowledge, the arthroscopic light bulb technique through outside-in fashion without distraction has not been reported in early-stage ONFH. The case report aims to present the case of a 29-year-old patient, who underwent the arthroscopic light bulb technique through outside-in fashion without distraction for bilateral ONFH, with an early outcome.

## Case presentation

**Figure 1:**
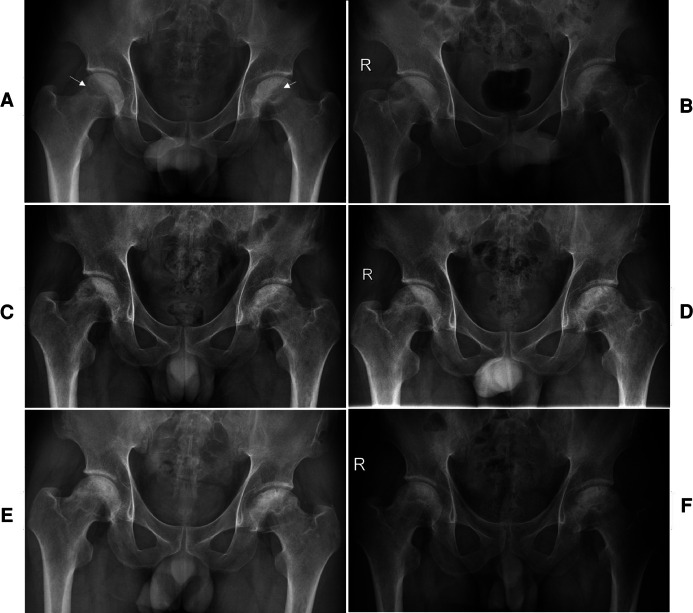
The preoperative x-ray shows cystic and sclerotic changes in the bilateral femoral heads (white arrow) (**A**). A series of postoperative x-ray (**B**, immediate), (**C**, 3 months), (**D**, 6 months), (**E**, 12 months), and (**F**, 24 months) show a gradual healing of the necrotic area at the bilateral femoral heads.

A 29-year-old male salesman complained of hip pain and limited range of motion (ROM) for 4 months in both hips. On July 10, 2019, insidious pain began in his left hip after walking, and the pain gradually subsided over 1 week without any treatment. At left hip pain relief after 1 month, the pain gradually started in both hips. The pain was alleviated after rest and aggravated with walking. No infectious contacts, such as tuberculosis, were found at his presentation. On primary physical examination, no warmth or swelling was noted in the patient's hip, nor was there any inguinal lymphadenopathy. X-rays of the pelvis showed osteoporosis of the femoral head on both sides (Figure 1A). The blood draw result was normal. He had an 11-year history of liquor intake in excess of 130 g per day and no history of glucocorticoid intake or trauma. No history of hereditary disease was found in his family. He was diagnosed with a synovitis of the hip and was managed with conservative methods such as the administration of non-steroidal anti-inflammatory drugs (NSAIDs), physical therapy, and functional exercises.

**Figure 2:**
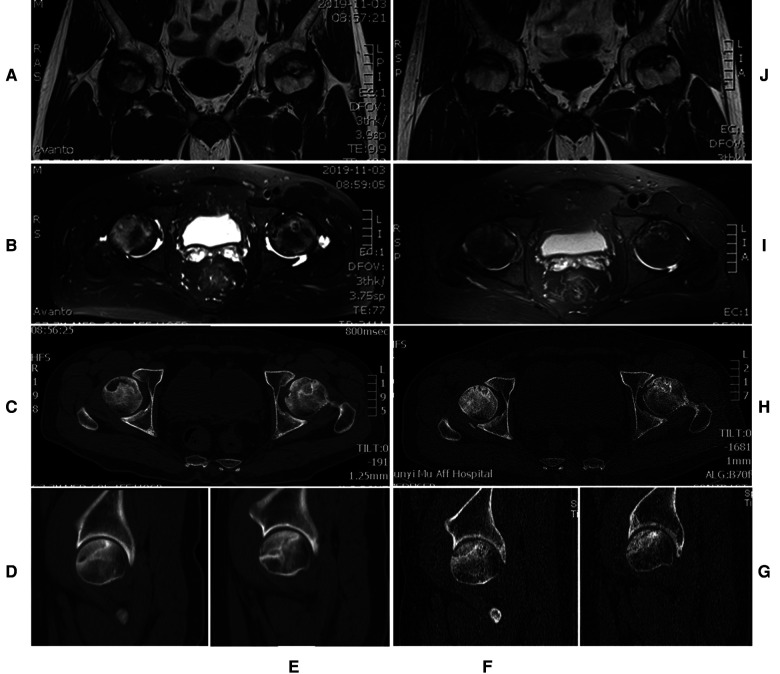
The preoperative axial MRI shows an area of a low-intensity signal on a T1-weighted image (**A**) and a high-intensity signal on a T2-weighted image (**B**). The preoperative axial (**C**) and sagittal (**D**, right side) (**E**, left side) CT scans show cystic and sclerotic changes at the anterosuperior area of the bilateral femoral heads. Postoperative MRI on T1-weighted (**J**) and T2-weighted (**I**) images show a decrease of cysts and oedema in the bilateral femoral heads. The postoperative axial (**H**) and sagittal (**F**, right side) (**G**, left side) CT scans show that the necrotic site is partly replaced by a new bone.

Bilateral hip pain and loss of ROM were gradually exacerbated by non-operative measures. Subsequently, he was admitted to the Department of Orthopedic Surgery of the Affiliated Hospital of Zunyi Medical University. Physical examinations showed limitations in weight-bearing during walking, and pain was noted in all planes of the hip by 15°. Preoperative Harris hip scores (HHSs) were 58 and 61 for the left and right hip, respectively. The Visual Analogue Scores (VAS) were 8 and 7 for the left and right hip, respectively. The patient showed limited ability to perform daily activities (Table 1). Magnetic resonance imaging (MRI) revealed geographically aberrant signal intensity impairments with typical appearances of bilateral ONFH (Figures 2A,B). Computed tomography (CT) (Figures 2C–E) showed a mixed appearance, including sclerosis and cysts on the bilateral femoral heads without flattening or crescent signs. The preoperative x-ray showed cystic and sclerotic changes in the bilateral femoral heads (Figure 1A). Laboratory test results were normal. He was diagnosed with bilateral ONFH (stage II) according to the Ficat classification (8). An all-arthroscopic light bulb technique through outside-in fashion without distraction (9) was recommended because no concomitant central compartment pathologies such as a labral tear, femoroacetabular impingement, and loose bodies were found on x-ray, CT, and MRI examinations.

**Table 1:** Scores of the hip preoperatively and at postoperative follow-up.

	Pre		Post 6 M		Post 12 M		Post 24 M	
	Left	Right	Left	Right	Left	Right	Left	Right
VAS	8	7	0	0	0	0	0	0
HHS	58	61	78	82	85	88	89	95
HOS-ADL	63.2	60.3	82.4	85.3	86.8	88.2	89.7	92.6
iHOT-12	39.2	41.7	77.5	79.2	80.8	82.5	82.5	84.1
Extension	0°	0°	8°	10°	11°	13°	13°	15°
Flexion	110°	110°	118°	120°	120°	122°	123°	125°
Adduction	5°	5°	15°	18°	18°	22°	22°	25°
Abduction	30°	30°	39°	40°	42°	42°	43°	45°
IR	30°	30°	37°	38°	40°	42°	43°	45°
ER	30°	30°	38°	40°	42°	43°	45°	45°

Pre, preoperative; Post, postoperative; M, months; VAS, Visual Analogue Score; HHSs, Harris hip scores; HOS ADL, Hip Outcome Score Activities of Daily Living subscale; iHOT-12, International Hip Outcome Tool-12; IR, internal rotation; ER, external rotation.

**Figure 3:**
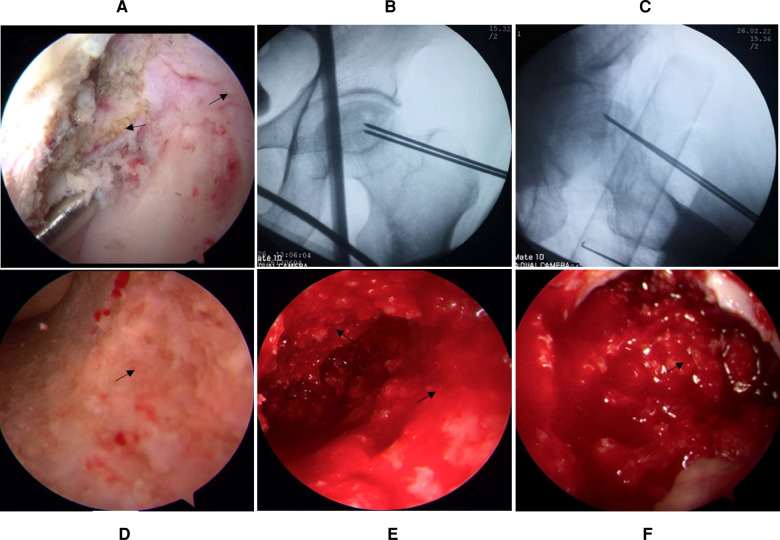
The capsule is cut longitudinally (the black arrow), and the necrotic site is located using a Kirschner pin under arthroscopic (**A**) and radiographic (**B,C**) visualization. (**D**) Appearance of the necrotic site before debridement. (**E**) Appearance of the necrotic site after debridement. (**F**) Arthroscopic appearance after bone particle grafting.

Arthroscopy was performed on the patient's left hip under general anesthesia through the anterolateral (AL) portal, distal anterolateral accessory (DALA) portal, and mid-anterior (MA) portal in the supine position on the traction table without applying distraction. The all-arthroscopic light bulb technique to the hip was applied according to outside-in fashion (10) and “touch method” (11). The AL portal was made after making a vertical stab incision, and the subcutaneous and muscular layers were split by using a straight clamp. The straight clamp was directed medially, pointing in the direction of the femoral neck. When the tip of the straight clamp touched the bone, it was replaced with a blunt trocar and the femoral neck could be felt, and the tip of the trocar was situated extra-articularly at the femoral neck of the hip joint. The DALA portal was made after making the skin incision, and a straight clamp was introduced and directed toward the arthroscope trocar. When the straight clamp touched the trocar of the arthroscope, the trocar was used as a guide to travel medially in the direction of the femoral neck. The straight clamp must touch the arthroscope trocar until the straight clamp touches the bone, and an ablator or shaver was introduced. After the fatty tissue of the precapsule was removed with the shaver, the white structure of the joint capsule was shown. Then, an extra-articular longitudinal capsulotomy was performed from outside to inside. The necrotic site was located using a Kirschner pin under arthroscopic (Figure 3A) and radiographic (Figures 3B,C) visualization before managing ONFH. Then, the MA portal was established under direct visualization.

After the fluoroscopic image of the Kirschner pin location was obtained to find the center of the necrotic site at the femoral head, a cortical bone window with a diameter of approximately 1.5 cm was established at the femoral head–neck junction with a cannulated drill through the DALA portal, and the necrotic bone (Figure 3D) was removed using a rongeur and curette until bleeding or healthy bone (Figure 3E) was found. Then, an arthroscopic check was performed to ensure that the necrotic bone was removed. Furthermore, the femoral head and neck were percutaneously penetrated for improving blood circulation using a 2.0 mm Kirschner pin (12). Finally, the ipsilateral auto-iliac cancellous bone was harvested and was used to fill the cavity of the femoral head (Figure 3F) through the cannula after necrotic bone removal. The surgery lasted 136 min. Two weeks postoperatively, the same procedure was performed on his right hip. The patient was followed up at 1, 3, 6, and 12 months and annually thereafter.

**Figure 4:**
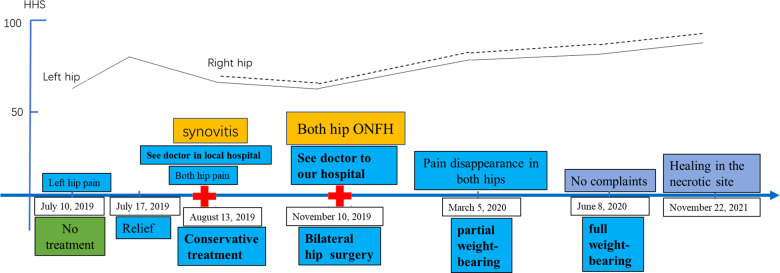
Osteonecrosis of the femoral head course and management response. The timeline from presentation detailing the preoperative Harris hip score response to surgery.

Early core-strengthening exercises and closed-chain hip stabilization were carried out to optimize the joint ROM (13). Relative to the literature (14, 15), in our institution, weight-bearing to prevent femoral head collapse is implemented with caution. For example, in the case under study, the patient was not allowed weight-bearing for the initial 3 months. Toe-touch weight-bearing using crutches was allowed for the second 3-month phase. The patient began full weight-bearing to prevent femoral head collapse when the grafted bone site fully healed (1). Three months after the procedure, the patient experienced no pain and had an improved VAS of 0. The VAS, HHSs, Hip Outcome Score Activities of Daily Living subscale (HOS ADL), International Hip Outcome Tool-12 (iHOT-12), and active ROM of the left hip and right side demonstrated consistent improvement at the 2-year postoperative follow-up (Table 1). This consistent improvement in outcomes showed that an all-arthroscopic light bulb technique through outside-in fashion without distraction could be a feasible method for the treatment of ONFH. The MRI (Figure 2I,J), CT (Figure 2F-H), and x-ray (Figures 1B–F) revealed a well-healed area of previous bone graft in the bilateral femoral heads, but there was radiological progress (slight collapse) on the left hip (Figures 1A–D). The patient was followed regularly and was strictly required to comply with the therapeutic plan. The patient presented that he was very satisfied at a follow-up visit 2 years after surgery. “As far as I know, ONFH is not always successful in hip-preserving procedure. The bilateral necrotic areas are healed, I am very lucky. The arthroscopic procedure is minimally invasive and aesthetically pleasing,” the patient said. Also, he did not have any complications. The timeline for ONFH course and management response was presented in Figure 4.

## Discussion

ONFH is a debilitating disease that, without effective and timely intervention, progresses to a collapse of the femoral head and secondary hip osteoarthritis, and the lack of timely intervention is the third main cause of THA (16). Multiple revisions are highly likely in young patients because of the average 90% longevity of two decades in most prostheses (17). Therefore, hip-preserving treatment is necessary to delay and/or avoid THA in young patients with early-stage ONFH (1). Early accurate diagnosis of ONFH is of great importance for the selection of therapeutic methods and prognosis. Early-stage ONFH is often atypical on x-rays and usually presents as mild osteoporosis, which could easily be misdiagnosed as synovitis of the hip. Transient osteoporosis of the femoral head is controversial as an early-stage of ONFH or a self-limiting disease based on reflex dystrophy. Core decompression is considered an effective method for diagnosing early-stage ONFH and transient osteoporosis (18, 19). In our case, an MRI investigation was considered important for early-stage diagnosis of ONFH, and this investigation revealed an area of a low-intensity signal on a T1-weighted image and that of a high-intensity signal on a T2-weighted image. MRI is considered the gold standard for detecting early-stage ONFH, with a sensitivity of more than 99% (19). A subchondral fracture shows a high T2-weighted signal on MRI but is usually obscured by joint effusion and bone marrow edema (BME). The subchondral fracture can also be underdiagnosed on an x-ray film (20). CT mainly focuses on the application of detecting subchondral fractures in early-stage ONFH. CT often shows sites of osteosclerosis surrounding the dead bone and new bone formation or reveals subchondral bone fracture (20, 21). When hip-preserving management is considered for early-stage ONFH, a CT examination is necessary for more accurate staging of ONFH (22). The mid-term and terminal stage ONFHs have typical manifestations on x-ray, CT, and MRI. This stage typically presents with subchondral fractures, femoral head collapse, secondary osteoarthritis, and acetabular degeneration (20, 21, 23).

The pathogenesis of ONFH is not completely clear, but the common characteristic of ONFH is the impaired blood supply of the femoral head (24). Therefore, hip-preserving treatment of ONFH includes several important principles such as debriding the necrotic bone, promoting revascularization and new bone formation, decreasing the intraosseous pressure of the femoral head and improving the venous circulation, providing the mechanical support of the femoral head, and preventing or halting femoral head collapse (25).

At present, various bone grafting surgeries are widely popular for the precollapse stage ONFH, which include vascularized and non-vascularized bone grafting. However, the various methods of bone graft vary (between 5.3% and 36.7%) in terms of failure incidence (2, 26). The incidence of failure is higher when it is an open or non-vascularized bone graft (14) than a close or vascularized bone graft (2, 16, 27, 28). Failure could be related to further impairment of blood supply (29–31). Therefore, minimally invasive arthroscopic surgery was chosen for our patient. We chose arthroscopic surgery for the following reasons: First, the outside-in fashion technique without distraction can avoid distraction- and perforation-related complications and save operating time due to the non-requirement for installing a traction table (9). Second, the arthroscopic procedure had been performed on a patient in a previous case report (6). Third, we perform this procedure on 60 patients per year on average. Fourth, based on mastering the outside-in fashion technique, we combined this with the “touch method” for posterior ankle arthroscopy (11), which further increased the accuracy and safety of surgery. Fifth, we employ the open bulb technique in 40 patients on average per year, which ensures the completion of surgery after it cannot be completed under arthroscopy. Sixth, the minimally invasive procedure reduces further injury to the blood supply and soft tissue.

Guadilla et al. (6) reported that the arthroscopic light bulb technique with distraction was used in three patients with early-stage ONFH. These patients achieved clinical improvement and radiological healing of the bone graft zone. In our case, similar results were obtained: 3 months after surgery, it was found that the patient was free of pain after a measurement of VAS, and after this, consistently improved ROM, HHSs, HOS-ADL, and iHOT-12 were obtained. At the 2-year postoperative follow-up, MRI, CT, and a series of x-rays revealed a well-healed area of previous bone graft in the bilateral femoral heads. These outcomes showed that the arthroscopic light bulb technique without distraction was a feasible method for the treatment of early-stage ONFH. The main reasons for the success and feasibility of this method lay in the protection of blood supply, removal of the necrotic bone, and effective support of the bone graft, all of which were minimally invasive. Good patient compliance was also an important factor. The process of moving from a no-weight-bearing to a full-weight-bearing stage after surgery can be a long and tedious one (6 months). Within a period of several weeks after grafting, cortical bone osteolysis occurs, which makes the graft weaker and decreases the support for the articular surface (32). Improper weight-bearing before the necrotic area has healed may lead to a collapse of the femoral head, which can mean a failure of the operation, and therefore, good patient compliance is an important factor. This case study indicated a significant improvement in the operative methods in the treatment of early-stage ONFH through an all-arthroscopic light bulb technique and outside-in fashion without distraction. This also highlights the fact that the arthroscopic light bulb technique through inside-out fashion with distraction is not an absolute requirement any longer (6).

Distraction- and perforation-related complications were not reported as a consequence of the arthroscopic procedure (6), and this may be related to the small sample size of cases and the performance of the operation by an experienced surgeon. The previous literature reported that the incidence rate of hip arthroscopy complications varied from 0.5% to 8%, and most of them were traction-related (10). Frandsen et al. (33) found that the complication rate of distraction-related injuries was up to 74% in hip arthroscopy. Therefore, distraction-related complications may not be completely avoided. In this case, we presented a new surgical approach that accomplished an all-arthroscopic light bulb procedure in outside-in fashion without distraction, which had never been reported in the literature previously. So far, the outside-in fashion method has been used for the treatment of central-compartment pathologies and THA-related complications (9, 10). This procedure establishes a manual space anterior to the hip joint capsule. Then, longitudinal capsulotomy is performed for the entry into the peripheral compartment without the need for distraction (34). The hardest step in performing this procedure on our patient was the orientation of the outside-in fashion for establishing a manual space anterior to the hip joint capsule. We applied the “touch method” for posterior ankle arthroscopy to solve the dilemma (11), which further increased the accuracy and safety of trocar and instrument localization by avoiding distraction- and perforation-related complications. In our case, the results showed that the treatment of early-stage ONFH by the arthroscopic bulb technique can be successfully performed without distraction, and complications can be avoided. In our experience, outside-in fashion without distraction reduced postoperative pain, radiation exposure, and tissue damage, prevented groin numbness, pudendal neurapraxia, and sciatic paralysis, and eliminated iatrogenic cartilage scufﬁng and penetration of the labrum during portal establishment. Longitudinal capsulotomy reduced the strength of the iliofemoral ligament and achieved good postoperative healing of the capsule and affected the stability of the hip joint less.

In this case, our all-arthroscopic light bulb technique proved to be a feasible method because the necrotic lesion was located at the anterosuperior site and no central compartment pathologies were found. However, the indications for the use of this technique are particularly limited, especially when it comes to the larger necrotic extent because osteonecrosis is located at the posterior site of the femoral head, leading to a higher-stage ONFH (≥Ficat stage III). Significantly, due to the limited space for arthroscopic surgery, when the dead bone was removed to have abundant bleeding or healthy bone at the track wall, the procedure of the dead bone removal can be stopped (6, 12). The extent of removal should be confirmed by hip arthroscopy (Figure 3E) or under radiographic fluoroscopy (15). Bone grafting procedures do not always require that the abnormal bone (such as the sclerotic bone) be completely removed (12). Extensive removal may not prevent removing the partially healthy bone and affecting the support of the femoral head (12). Lastly, weight-bearing should be implemented with caution after surgery. The necrotic area will undergo partial resorption of the bone graft and then complete healing by crawling substitution. Duration of the healing, this weakens the support of the necrotic area to the articular surface (15, 32). To further evaluate the safety and effectiveness of our technique, it is necessary to carry out large-scale, prospective clinical studies in the future. However, there is a caveat. The all-arthroscopic light bulb technique through outside-in fashion without distraction may be performed by experienced and specialized arthroscopic surgeons. For beginners, the performance of this technique may consume more operation time compared with the open bulb procedure because hip arthroscopic surgery involves a learning curve (10, 35).

In conclusion, an all-arthroscopic light bulb technique through outside-in fashion without distraction could be a feasible method for the treatment of ONFH. This case highlights the fact that distraction- and perforation-related complications can be avoided in patients with ONFH without the concomitant pathologies of the central compartment.

## Data Availability

The original contributions presented in the study are included in the article/Supplementary Material, further inquiries can be directed to the corresponding author.
